# Developing a list of core competencies for medical aspects of healthcare delivery in care homes: scoping review and Delphi process

**DOI:** 10.1093/ageing/afad237

**Published:** 2023-12-28

**Authors:** Lauren McCarthy, Kayla Borley, Thomas Ancelin, Rachael Carroll, Neil Chadborn, Adrian G Blundell, Adam L Gordon

**Affiliations:** Academic Unit of Injury, Recovery and Inflammation Sciences (IRIS), School of Medicine, Nottingham, UK; Academic Unit of Injury, Recovery and Inflammation Sciences (IRIS), School of Medicine, Nottingham, UK; Academic Unit of Injury, Recovery and Inflammation Sciences (IRIS), School of Medicine, Nottingham, UK; Academic Unit of Injury, Recovery and Inflammation Sciences (IRIS), School of Medicine, Nottingham, UK; NIHR Applied Research Collaboration – East Midlands (ARC-EM), Nottingham, UK; Academic Unit of Injury, Recovery and Inflammation Sciences (IRIS), School of Medicine, Nottingham, UK; NIHR Applied Research Collaboration – East Midlands (ARC-EM), Nottingham, UK; Academic Unit of Injury, Recovery and Inflammation Sciences (IRIS), School of Medicine, Nottingham, UK; Department of Health Care of Older People, Nottingham University Hospitals, Nottingham, UK; Academic Unit of Injury, Recovery and Inflammation Sciences (IRIS), School of Medicine, Nottingham, UK; NIHR Applied Research Collaboration – East Midlands (ARC-EM), Nottingham, UK; Department of Medicine of the Elderly, University Hospitals of Derby and Burton NHS Foundation Trust, Derby, UK

**Keywords:** care homes, homes for the aged, graduate medical education, primary care, qualitative research, older people

## Abstract

**Background:**

Care home residents live with frailty and multiple long-term conditions. Their medical management is complex and specialised. We set out to develop a list of core competencies for doctors providing medical care in long-term care homes.

**Methods:**

A scoping review searched MEDLINE, EMBASE and CAB Abstracts, supplemented by grey literature from the Portal of Online Geriatrics Education and the International Association of Geriatrics and Gerontology, looking for core competencies for doctors working in care homes. These were mapped to the UK nationally mandated Generic Professional Competencies Framework. A Delphi exercise was conducted over three rounds using a panel of experts in care homes and medicine of older people. Competencies achieving 80% agreement for inclusion/exclusion were rejected/accepted, respectively.

**Results:**

The scoping review identified 22 articles for inclusion, yielding 124 competencies over 21 domains. The Delphi panel comprised 23 experts, including 6 geriatricians, 4 nurses, 3 general practitioners, 2 advanced clinical practitioners, 2 care home managers, and one each of a patient and public representative, palliative care specialist, psychiatrist, academic, physiotherapist and care home audit lead. At the end of three rounds, 109 competencies over 19 domains were agreed. Agreement was strongest for generic competencies around frailty and weaker for sub-specialist knowledge about specific conditions and competencies related to care home medical leadership and management.

**Conclusion:**

The resulting competencies provide the basis of a curriculum for doctors working in long-term care homes for older people. They are specialty agnostic and could be used to train general practitioners or medical specialty doctors.

## Key Points

- Older people living in care homes have complex medical care needs.
- Medical care for older care home residents in most countries is provided by doctors without specific training in the sector.
- This paper outlines 109 learning outcomes over 19 domains as a basis of postgraduate education of doctors working in care homes.
- These findings could also inform incorporation of learning outcomes around care homes into undergraduate curricula.

## Background

Care homes provide 24-hour care, with or without nursing, for people who have disability and dependency that cannot be met by care at home. There are specific care homes for people with learning disabilities and mental health conditions. The majority of care homes, however, are registered for care of older people. In the UK, there are around 11,000 care homes, providing care for around 410,000 people [1]. The projected expansion in the older population, with associated increases in the population prevalence of frailty, dementia and long-term conditions, are projected to offset technologies enabling care closer to home, such that the care home sector will remain at its current size, or slightly larger, until the middle of this century [1]. Knowing how to care for care home residents will therefore be important for doctors for the foreseeable future.

The average care home resident is over 80, has multiple diagnoses, takes multiple medications and requires help with activities of daily living [2]. Many are near the end of life. Around 70% have dementia or cognitive impairment [2]. In the UK, national policy initiatives have recognised the complexity of older people living in care homes through contract arrangements that incentivise more frequent visiting by general practitioners and which aim to reinforce access to rehabilitation, mental health and palliative care expertise [3].

The processes that underpin effective multidisciplinary care in care homes are increasingly well described [4]. Most countries, however, have no agreed standards for medical practice in care homes [5]. Only in the Netherlands, where the specialty of Elderly Care Medicine developed and grew from the nursing home sector, is there a medical specialty with a comprehensive curriculum specifically focussed on the competencies required to meet the day-to-day needs of the care home population [6]. In UK postgraduate training [such as the General Practice (GP) or geriatric medicine specialty curricula], there is little mention of delivery of healthcare in care homes, over and above the content related to more generic care of older people with frailty, multimorbidity and dementia [7, 8]. An interview study of senior and trainee GPs found limited training in the field, whilst the diversity of organisation of both GPs and care homes meant that it was difficult to teach a ‘standard’ way of working [9].

Against this background, we set out to describe a set of core competencies for doctors providing healthcare to older people living in care homes. We recognised that this type of care is provided by different specialties in different places, and often by teams of doctors—general practitioners, geriatricians, old age psychiatrists and palliative care physicians—with overlapping expertise, each of whom can meet part of a care home resident’s care needs. With this in mind, we aimed to describe the competencies required to meet the needs of care home residents in a specialty-agnostic way, rather than starting from the perspective of one medical discipline.

## Methods

We approached the work in two stages. First, we conducted a scoping literature review to identify publications that described core competencies for doctors working in care homes—synthesising these to summarise domains and ensure consistency of language. Second, we conducted a Delphi consensus exercise to shape these into a definitive list of competencies.

The Delphi process began in 2019, prior to the COVID-19 pandemic, was aborted in 2020 because the study team were redeployed to support clinical care during the pandemic, and then recommenced in 2022.

### Stage 1—scoping review

The literature review protocol has been published on the Open Science Framework [10]. Our work is reported here in line with PRISMA-ScR guidelines [11]—a PRISMA-ScR checklist is provided in Supplementary Appendix 1.

The objective of the review was to identify and describe competencies for doctors providing healthcare to care home residents—or residents of equivalent institutions in other countries—which then could provide the basis of a subsequent Delphi process.

Initial searches were conducted in September 2019 prior to the COVID-19 pandemic, but were refreshed in 2022 to ensure that no new important literature had emerged in the interim that would substantively change the consensus work that followed. We report the search strategy for the second search in order to convey the latest date up to which we searched the literature. We searched EMBASE (1974 to 2022 Week 46), MEDLINE (1946 to 22 November 2022) and CAB abstracts (1910 to 2022 Week 46). Search terms are summarised in Table 1 and an exemplar search string from MEDLINE is outlined in Supplementary Appendix 2. Additional grey literature searches were conducted using the Portal of Online Geriatrics Education and the website of the International Association of Geriatrics and Gerontology.

**Table 1:** Search terms

1st term	Core competencies	Postgraduate	Care home
Broader terms and synonyms	Clinical competence Curricula^*^.^*^ Education Competency-based education Educational measurement Teaching	Geriatric^*^.^*^ Gerontology Health services for aged Physician	Nursing homes Homes for the aged Residential facilities Long-term care Aged Aged, 80 and over Frail elderly

^*^.^*^ indicates wildcard used to expand the search term

We included English-language articles that described curricula, teaching, standards, assessment or competencies; at a postgraduate level; and directly related to care delivery in care homes or an equivalent setting. All study designs were included. We excluded articles describing competencies of non-medical staff; undergraduate medical education; and disease-specific competencies; conducted in care homes for children, those with learning difficulties, rehabilitation facilities, prison, temporary accommodation or any other intermediate care settings or hospice facilities. Articles were screened for inclusion at each of title, abstract and full text stage by two reviewers (KB/LM), erring in favour of inclusiveness. Where inclusion was uncertain, articles were discussed with the full authorship group. We did not undertake a quality appraisal since the primary function of this process was a scoping exercise to identify competencies that could be carried forward to the Delphi process in stage 2. Data extraction focussed mainly upon extracting published competencies from the literature, but a data extraction template also included data on the year and country of publication, and article type (curriculum, review article, editorial) to provide context.

A synthesis process involved the full authorship group looking at the competencies together. The extracted competencies were then condensed and reworded by categorising into ‘no change’, ‘merge’, ‘delete due to duplication’ and ‘reword’. Rewording was to ensure competencies were applicable to the UK context. To aid this rewording and further contextualise the competencies in a way that would facilitate the subsequent Delphi process, they were categorised against the domains of the Generic Professional Capabilities Framework (GPCF)—produced by the General Medical Council, the national registration body for doctors, and designed to express the principles and professional responsibilities of doctors as competencies [12]. We ensured that wording was pedagogically sound by rewording with reference to Bloom’s taxonomy [13]. First published in 1956, Bloom’s taxonomy laid out cognitive (knowledge), psychomotor (manual skills) and affective (attitudes) domains, enabling learning outcomes to be expressed in a way that could easily be taught and assessed. It remains a dominant theory, driving practice in medical education over half a century later [14].

### Stage 2—Delphi exercise

Competencies derived from the literature review were carried forward to a Delphi process. The Delphi method has been used across various fields of healthcare research including policy change, resource utilisation and for preliminary work to develop curricula [15, 16]. The objective for this stage of work was to establish a set of learning objectives for postgraduate training of doctors to deliver expert day-to-day care in a care home setting.

A panel of experts was convened to encompass a range of views of the competencies required by medical practitioners day-to-day in care homes. We aimed to recruit experts in frailty, primary care, rehabilitation, old age psychiatry, palliative care and multidisciplinary working. We made an initial approach to members of the Community and Primary Care Group of the British Geriatrics Society (BGS). The BGS is the multidisciplinary specialist organisation representing UK professionals with an interest in care of older people, and the Community and Primary Care Group comprises a mix of community geriatricians, general practitioners (GPs), nurses and allied health professionals. We supplemented this with snowball sampling using introductions from members of this group. We recruited lay representatives from regularly scheduled Patient and Public Involvement groups convened by the University of Nottingham and focussed on care of older people.

Panel inclusion criteria were as follows: experience of working with older people with frailty and/or multiple long-term conditions within the care home setting OR; expertise in curriculum design around frailty, ageing and associated comorbidities OR; lay interest in and/or experience of the health care of older people living in care homes. Participants who were not fluent in written English were excluded on the basis that the process would be conducted online by email. In total, 34 people were approached for involvement.

Emails were sent to participants individually to ensure anonymity. At each round, participants were given 3 weeks to respond, with reminder emails sent weekly. If a participant had not completed the round at this point, with no contact with the researchers, they were excluded from further rounds of the process.

To ensure strong retention of expert involvement, an upper limit of three rounds of investigation was set in this study.

Each questionnaire consisted of a series of competencies that the respondent could rate as ‘include’, ‘exclude’ or ‘requires further development’. Where ‘requires further development’ was selected, a free-text box enabled respondents to suggest changes. In the first round, respondents were also asked whether additional competencies were required.

The Delphi process is an iterative process that uses repeated communication to refine expert opinion on the topic and move towards an accepted level of agreement. After each round:

- A summary of the panel scores was presented for each competency and the role description.
- Any statement that reached consensus was removed from further rounds.
- Some participants agreed competencies, but at the same time suggested changes to the wording of the text. Competencies where over 80% agreement was reached were modified where the free-text comments indicated this was appropriate. Where modifications were minor (i.e. to add clarity to the competency or use of more precise language), the amendments were highlighted; however, the panel was not asked to rescore. Other modifications were considered major, and the panel was asked to rescore.
- New competencies were formulated based on free-text comments.
- Competencies were combined or separated based on suggestions given.

There is no universally accepted threshold for defining consensus as part of the Delphi process. Consistent with other studies [15, 16], consensus was considered to have been reached when there was ≥ 80% percentage agreement to ‘include’ or ‘exclude’ between the panel members. We conducted three rounds. The first round took place in January 2020. The work was aborted due to the COVID-19 pandemic and recommenced in November 2022, when the second round took place. The third, and final, round took place in January 2023. Failure at the third round of the questionnaire to achieve an 80% consensus on inclusion or exclusion led to exclusion of the competency.

Ethical approval was not needed based upon advice from the University of Nottingham School of Medicine Research Committee and using online Health Research Authority decision-making tools.

## Results

### Literature review

**Figure 1:**
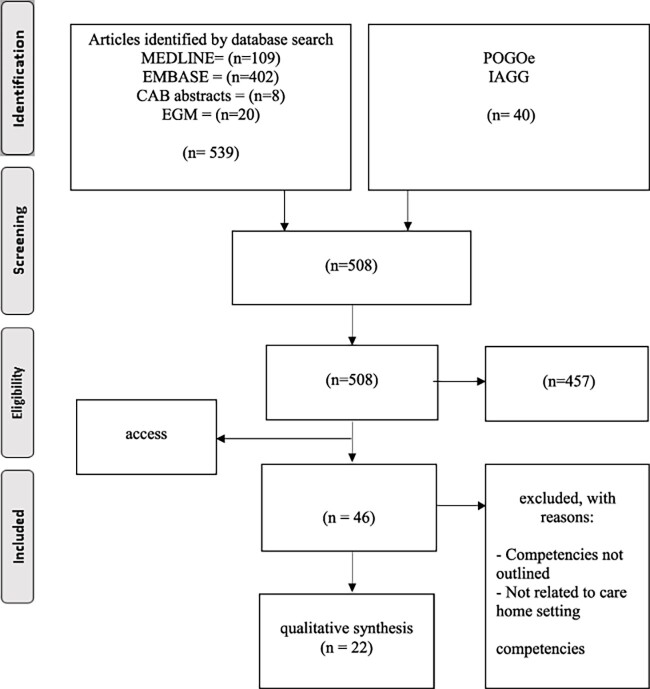
PRISMA diagram for scoping review.

For the literature review, 508 articles retrieved after de-duplication, of which 5 were inaccessible using available interlibrary loan services, and 457 were excluded at abstract screening. A further 24 were excluded at full article screening, leaving 22 for inclusion in the review. A full PRISMA diagram is shown in Figure 1.

Articles were published between 2002 and 2022. Seventeen articles came from the USA, two from Canada and two from Hong Kong, and one from the Netherlands. The bulk of the articles (*n* = 16) were peer-reviewed papers, two were conference abstracts, and one each comprised a non-peer-reviewed published curriculum, plenary lecture, position paper and a discussion paper. From these, we extracted 456 competencies. Following de-duplication and rewording, we were left with 61 competencies. The final list of competencies from the literature, mapped against the GPCF, is shown in Supplementary Appendix 3.

To enable easy deliberation on these competencies as part of the subsequent Delphi exercise, we broke these down into the smallest chunks possible, where each bullet-point referred to one item for inclusion/exclusion, and reorganised them into domains that enabled better categorisation of competencies otherwise grouped together under the second domain of the GPCF. This generated 124 competencies over 21 domains, summarised in Supplementary Appendix 4.

### Delphi process

The constitution of the Delphi panel is shown in Table 2. Twenty participants responded to the initial Delphi exercise in 2020, with 23 responding to the second round after the Delphi resumed in November 2022, and 23 respondents participating in the third round in January 2023.

**Table 2:** Delphi exercise participants

Geriatrician	6
Nurse	4
GP	3
Advanced clinical practitioner	2
Care home manager	2
Patient and public representative	1
Palliative care specialist	1
Psychiatrist	1
Academic	1
Physiotherapist	1
Audit lead	1

**Figure 2:**
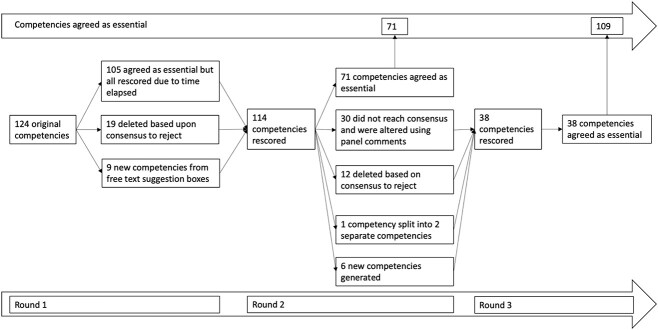
Flowchart of Delphi competencies through rounds 1–3.

A flowchart showing how competencies moved through the consensus process is shown in Figure 2. Because of the time-lag between the first and second rounds of Delphi due to the COVID-19 pandemic, competencies agreed as essential at the first round were tested again during the second round.

At the end of this process, 109 competencies were agreed across 19 domains; these are summarised in Table 3.

**Table 3:** Agreed competencies following Round 3 of Delphi Exercise

Domain	Competencies
**1. History-taking and communication with residents and their families**	1.1 Can take an appropriately focussed history to include residents with multiple long-term conditions and complex needs
	1.2 Can take a collateral history from both relatives and care staff to support relationship-centred care and shared decision-making
	1.3 Can communicate complex problem lists and management plans without using medical jargon to ensure residents, family and carers understand
	1.4 Can communicate effectively with people with cognitive impairment/communication difficulties including being able to recognise and assess when that person is in distress
	1.5 Can communicate effectively around sensitive topics such as advance care planning
	1.6 To be able to effectively elicit concerns from care home staff, taking account of their observations and feelings to develop a working patient history
	1.7 Can target history-taking to discriminate between likely diagnoses as part of a comprehensive approach to medical assessment
	1.8 Can assess capacity to give informed consent
	1.9 Can understand best interests and appropriate decision-making on behalf of families and carers
	1.10 Can recognise gender, sex and sexuality as part of holistic care in a care home setting
	1.11 Can identify limited health literacy of residents and their families as a barrier to communication and use strategies to overcome this
**2. Management of long-term conditions**	
2.1 Diabetes	2.1.1 Can describe the diagnosis, pathophysiology, management and preventative strategies for diabetes
2.2 Cardiovascular	2.2.1 Can describe the diagnosis, pathophysiology, management and preventative strategies for stroke
	2.2.2 Can effectively manage heart failure
	2.2.3 Can describe the diagnosis, pathophysiology, management and preventative strategies for ischaemic heart disease
2.3 Mental illness and wellbeing	2.3.1 Can recognise symptoms, undertake basic assessment and advise on management of mood and affect changes
	2.3.2 Can describe the diagnosis, management and preventative strategies for anxiety
	2.3.3 Can describe the diagnosis, management and preventative strategies for depression
	2.3.4 Can recognise symptoms, undertake basic assessment and advise on management of sleep issues
	2.3.5 Considers the role of lifestyle factors including alcohol and substance intake in the lives of care home residents and can screen for dependency where indicated
2.4 Neurodegenerative conditions	2.4.1 Can describe the diagnosis, pathophysiology, management and preventative strategies for Parkinsonism
	2.4.2 Can describe the diagnosis, pathophysiology and management of other common neurodegenerative conditions such as motor neurone disease and multiple sclerosis in conjunction with specialist support
2.5 Dementia	2.5.1 Can conduct a Cognitive Status Assessment
	2.5.2 Can assess and manage behavioural and psychological symptoms associated with dementia including behaviours that challenge in conjunction with specialist services
	2.5.3 Has knowledge of the effect of dementia on management of comorbidities
	2.5.4 Has knowledge of the commonly used drug treatments for dementia
2.6 Skin integrity	2.6.1 Can describe the diagnosis, pathophysiology and management of skin integrity issues including pressure ulcers in conjunction with nursing team and specialist tissue viability services
2.7 Cancer	2.7.1 Can effectively support the ongoing management of patients with cancer in collaboration with hospital-based oncologist and palliative care teams
2.8 Renal disease	2.8.1 Can describe the diagnosis, pathophysiology and management of chronic kidney disease including identifying need for specialist referral and/or palliative care
2.9 Peripheral vascular disease	2.9.1 Can describe the diagnosis, pathophysiology and management of peripheral vascular disease and know when to refer to specialists
2.10 Management of multiple long-term conditions	2.10.1 Can effectively manage patients with multiple long-term conditions
	2.10.2 Can individualise standard recommendations for screening tests and chemoprophylaxis in older patients based on life expectancy, functional status, patient preference and goals of care
**3. Management of frailty syndromes**	
3.1 Delirium	3.1.1 Can recognise, diagnose and manage delirium in combination with non-specific presentations of illness presenting both acutely or sub-acutely
3.2 Continence	3.2.1 Can assess and manage urinary and faecal incontinence in liaison with the continence team
	3.2.2 Knows how and when to refer for further specialist advice (e.g. continence nurse specialist, OT), appreciating that referral pathways will differ locally
	3.2.3 Can act on advice and support decision-making in the management of urethral, suprapubic catheters and urethral sheaths, as well as knowing when to insert and when to initiate a trial without catheter
	3.2.4 Can provide advice on medical aspects of continence optimisation and preservation as part of a wider multidisciplinary team
3.3 Medication side effects	3.3.1 Can describe changes in the pharmacodynamics and pharmacokinetics of commonly used drugs within the older population
	3.3.2 Can recognise the importance of drug–patient and drug–drug interactions in this cohort
	3.3.3 Can make appropriate prescribing and de-prescribing decisions, including the use of structured tools (e.g. STOPP/START) when appropriate
	3.3.4 Understands the clinical consequences of polypharmacy in the care home setting
	3.3.5 Is able to communicate key information on risks/adverse outcomes from prescribing to staff without nursing or medical qualifications
3.4 Falls	3.4.1 Has knowledge of interventions for falls prevention
	3.4.2 Is able to take a history for falls and examine for common causes of falls
	3.4.3 Is able to perform clinical assessment of injuries to identify residents in need for further investigation/imaging
	3.4.4 Understands principles of positive risk management—risks can sometimes be mitigated but not averted
3.5 Immobility	3.5.1 Can screen for risk of fractures using risk tool such as FRAX
	3.5.2 Can support the management of osteoporosis using evidence-based treatments
	3.5.3 Can diagnose and manage osteoarthritis
	3.5.4 Can examine gait and balance and refer for multidisciplinary assessment when indicated
	3.5.5 Can effectively manage musculoskeletal problems
**4. Management of sensory impairments**	4.1 Can recognise symptoms, undertake basic assessment and refer where appropriate for visual impairments
	4.2 Can recognise symptoms, undertake basic assessment and refer where appropriate for hearing impairments
**5. Knowledge of ageing**	5.1 Has knowledge of the functional, physical, cognitive, psychological social and spiritual changes common in older age
	5.2 Can conduct a Comprehensive Geriatric Assessment
	5.3 Recognises that clinical uncertainty is common in care home residents and be able to manage this uncertainty in prognostication and care conversations
**6. Nutrition**	6.1 Can screen for nutritional deficiency and refer for specialist input as appropriate
	6.2 Can work with other health professionals to devise appropriate nutritional support strategies for residents
	6.3 Can work with the wider multidisciplinary team to modify nutritional approaches to take account of disease processes, tissue viability, recovery from illness and surgery
	6.4 Can support decision-making around oral nutritional supplementation and tube feeding where appropriate
**7. Rehabilitation**	7.1 Can establish which disabilities may be amenable to rehabilitation alongside therapy and rehabilitation medicine professionals
	7.2 Can provide medical support to a multidisciplinary team in developing rehabilitation plans based upon a resident’s health, wellbeing, person-centred goals and motivation
**8. Management of acute conditions**	8.1 Can identify an acutely unwell patient and collaboratively make decisions about escalation of treatment, including hospital transfer
	8.2 Has knowledge of monitoring acute conditions by making effective use of RESTORE2 or other appropriate early-warning assessment tools
	8.3 Can appropriately prioritise acute conditions over less urgent clinical problems
	8.4 Can recognise the signs and symptoms of overdose in drugs commonly used in care homes and consider how presentation may be affected by cognitive impairment and physical disability
	8.5 Can consider how undifferentiated illness can be a manifestation of diseases that often present atypically in older adults (e.g. acute coronary syndromes, the acute abdomen, urinary tract infection and pneumonia)
**9. End-of-life care**	9.1 Can recognise when to discontinue investigations and disease-modifying treatment on an individualised, holistic and situationally appropriate basis
	9.2 Can recognise when to prescribe anticipatory medications
	9.3 Can manage common ethical dilemmas in residents including resuscitation and escalation of treatment, including when residents lack capacity to make these decisions
	9.4 Can describe and use care pathways to support care of the dying, such as the Gold Standards Framework and NICE guidelines for last days of life
	9.5 Can recognise when to seek specialist palliative care input
	9.6 Has knowledge and clinical expertise in palliative and end-of-life care in dementia
**10. Pain management**	10.1 Knows how to assess pain in adults with frailty and cognitive impairment
	10.2 Is able to modify pain management strategies in residents with communication difficulties and cognitive impairment using non-pharmacological strategies
	10.3 Is able to modify pain management strategies in residents with communication difficulties and cognitive impairment using modifications to common analgesic regimens
	10.4 Can provide advice and support around a range of palliative care medications, including syringe drivers, in the care home setting. Knows when to seek help from a specialist
	10.5 Is aware of appropriate delivery mechanisms for analgesia
	10.6 Can distinguish between different types of pain (e.g. neuropathic and non-neuropathic) and modify treatments accordingly
	10.7 Is able to appropriately prescribe regular PRN analgesia for patients who will not prompt for pain relief
**11. Therapeutics and safe prescribing**	11.1 Can describe a range of approaches to support medication adherence in older patients, for example, by using compliance aids, changing preparations and dosing schedules
	11.2 Has an understanding of the challenges and dilemmas in covert administration of medication for patients without mental capacity
	11.3 Can work with pharmacy and speech and language therapy colleagues to advise on the appropriate use of topical, crushed, dispersible, liquid and transdermal medications
**12. Infection control**	12.1 Can describe the specific challenges of infection control in the care home sector
	12.2 Can describe how to manage communicable diseases to include diarrhoea/vomiting, scabies and respiratory illnesses including influenza and coronaviruses in the care home sector
	12.3 Can describe prevention vaccination strategies required to support health and wellbeing in the care home sector
**13. Communication and cooperation with colleagues**	13.1 Can communicate with staff with and without nursing qualifications, maintaining confidentiality
	13.2 Is aware of recent developments around electronic care planning in care homes and can use this understanding to develop a shared approach to record keeping with care home teams
	13.3 Is able to support care home staff in the initiation of sensitive or challenging conversations with residents and/or their families
**14. Working collaboratively with care homes**	14.1 Can describe the staffing structures within modern care homes and recognise the expertise, competencies, roles and responsibilities held by different team members
	14.2 Is able to recognise the complementary roles and responsibilities of health and social care staff in providing care
	14.3 Can describe the ways in which doctors can support care home teams to deliver routine aspects of healthcare
**15. Teaching and training**	15.1 Can support learning and development of the care home workforce both formally and informally, recognising the needs of diverse learners
**16. Quality improvement and evidence-based practice**	16.1 Is able to find and interpret current best evidence in long-term care
	16.2 Can participate as part of the wider multidisciplinary team around quality improvement in care homes
	16.3 Is able to recognise the barriers and challenges to quality improvement in the care home context
**17. Capabilities in safeguarding vulnerable groups**	17.1 Is able to recognise and respond to abuse of older people including physical, psychological, emotional, financial, sexual and institutional abuse
	17.2 Is able to describe safeguarding procedures and adhere to these
	17.3 Is able to support care staff who may raise safeguarding concerns regarding vulnerability or abuse
**18. Leadership team working**	18.1 Is able to listen, support and advise a team of professionals and care staff with diverse clinical and educational backgrounds
**19. Legal frameworks**	19.1 Can describe the care home specific considerations with regard to death certification and the role of the Coroner/Procurator Fiscal
	19.2 Can describe the care home specific considerations with regard to The Mental Health Act
	19.3 Can describe the care home specific considerations with regard to The Mental Capacity Act
	19.5 Can describe the care home specific considerations with regard to Deprivation of Liberty Safeguards
	19.6 Can describe the care home specific considerations with regard to confidentiality
	19.7 Can describe the care home specific considerations with regard to advance directives and decisions
	19.8 Can describe the care home specific considerations with regard to Lasting power of attorney
	19.9 Can describe the care home specific considerations with regard to decisions regarding resuscitation
	19.10 Can describe the care home specific considerations with regard to communicable disease notification

Key areas of disagreement between respondents during the Delphi process related to (i) the extent to which condition-by-condition competencies should be outlined in a curriculum; (ii) the extent to which doctors should be involved in administrative aspects of the care home pertaining to healthcare; and (iii) the balance between the care home medical practitioner as a competent generalist who knew when to refer on for advice, and as an expert practitioner in frailty, dementia and associated syndromes, who could manage complex and specialised issues in situ.

## Discussion

We conducted a literature review and Delphi process to outline core competencies for doctors working in UK care homes. Data extraction and analysis yielded 124 competencies across 21 domains. Literature review findings were the basis of a Delphi process that led to consensus on 109 competencies covering 19 domains.

The final domains range from history-taking and examination, through syndromes common in later life to aspects of teamwork and care home organisation relevant to medicine in a care home setting. Agreement was achieved most readily around competencies that involved the co-ordination of care in a complex group of patients—for example, in frailty, dementia and end-of-life care. Syndrome specific expertise, for example in Parkinson’s or motor neurone disease, was more contentious—in part because these were seen to be the domain of specialty doctors who already ‘own’ long-term conditions management for these groups. Agreement about organisational involvement of doctors in the running of care homes was limited to roles in teaching staff and supporting quality improvement/assurance around healthcare. This is different from the quite detailed requirements for Dutch Elderly Care Physicians [6] and US Medical Directors [17] to be educated to take on organisational leadership roles in long-term care organisations that employ them directly. This could be symptomatic of the fragmented nature of care delivery in the UK, with uncertain boundaries between health and social care, and public and private, providers [18]. An additional factor may be the well-reported under-resourcing of Primary Care in the UK that drives reluctance to advocate for such extended roles for doctors. Previous publications from the Netherlands and the USA have described competencies for Elderly Care Physicians [6] and nursing home Medical Directors [14], respectively. The work we have presented here is different in that it starts from a question bounded not by specialism or role, but from the perspective of what any doctor working routinely in care homes would need to know. This is important for two reasons. Firstly, it moves the discussion away from potentially territorial aspects about which specialty has the historical prerogative, capacity or will to undertake the work (‘who should be doing this?’), to the much more pragmatic and patient-centred perspective of what skills are required to deliver good care (‘what should be done?’). Secondly, it reflects the situation in most countries outside of the USA and the Netherlands, where the medical responsibility for care home residents is unclear, shared or disputed between different groups of practitioners [19]. The approach taken therefore represents a template for how doctors in other countries might approach this question. It lays the possible foundation for agreement of competencies across Europe as called for by the European Union Geriatric Medicine Society [5].

These competencies represent a conversation starter. Which doctors currently possess these skills, and which would be well placed to develop them going forward? How can healthcare delivery in care homes be structured to ensure that doctors with the right skills are available to residents and care home staff, at the right time? Does this mean that one specialty needs to train differently, or that *more* than one specialty needs to train differently? Or is there scope—as we see in some other countries—for a specialty, or sub-specialty, focussed on care of older people in care homes? A recent interview study indicated that GP training in these topics is ad hoc and the diverse practices means that learning is not standardised [9]. Although other medical specialties that work in care homes have not been studied in depth, their training in this space is likely to be similarly variable. These are issues for our national healthcare and health education policymakers, and for Royal Colleges. Supplementary Appendix 3—mapping the literature derived competencies to the Generic Professional Capabilities Framework—shows that the competencies we have derived here are ‘in scope’ for doctors. Which doctors they should be in scope for remains unclear. Across Europe and the rest of the world, this requires negotiation, reconfiguration and shared learning as different jurisdictions work out how to care for this under-supported group of people with complex health problems. The route from defining these competencies to changing frontline medical practice in care homes is potentially long and complex—involving implementation challenges at the individual, team and organisational level [20]. This work could potentially be delivered by stakeholder organisations, such as specialty societies, with links at policy (macro), planning (meso) and provider (micro) levels.

Part of the conversation should focus on what is missing from these competencies. The Delphi process is good at achieving consensus, but less effective at generating new ideas or expanding upon existing ones. An example is delirium, which is represented in the competencies predominantly with regard to identification and treatment. Yet, the increasingly robust evidence-base for delirium prevention interventions [21] might, arguably, be just as important in care homes. Well-written competencies are supposed to make implicit the underpinning knowledge, skills and attitudes—yet ageist attitudes can undermine effective education and training interventions around care of older people [22]. It may be that further iteration is required to better embed the attitudinal and behavioural aspects of good care for older people into the competencies proposed. Long-term care settings achieve limited attention as part of undergraduate medical syllabuses [23], and it is possible that the competencies presented here could provide the basis of introducing more teaching on these topics for medical students.

Good healthcare delivery in care homes is, of course, multi-professional in nature [3, 4]. Doctors work as part of a wider multidisciplinary team. A previous Delphi exercise focussing on competencies for nurses working in care homes agreed 22 competencies but, again, struggled with condition-specific ‘sub-specialty’ competencies and how ‘core’ leadership and management competencies should be considered to be for care home nurse [24]. This uncertainty over where the leadership for healthcare in care homes lies, reported elsewhere in the literature [17], makes it essential that competencies in multidisciplinary teamworking are included for all who work in the sector. Correlation and cross-referencing between any newly developed curricula for different professional groups who must work together is essential. It is possible that at least some of the same competencies around leadership and teamworking should and could be replicated across the curricula for multiple professions.

The strengths of this work relate to the structured approach taken to derive a long list of competencies from the published literature, and to the way in which these were synthesised to take account of prevailing approaches to health education and healthcare delivery in order to maximise the cogency of the subsequent Delphi exercise. The Delphi exercise recruited through a large and well-recognised national specialty organisation and recruited a broad range of professionals with a perspective on healthcare delivery in care homes. The limitations of the work relate to the fact that some curricula on healthcare delivery—most notably those from the Netherlands—are not published in English and could not be included. As with all Delphi exercises, the representativeness of the panel is a potential limitation. It is likely that the perspective of geriatricians is over-represented in the final recommendations. The legitimacy of this given that in many parts of the UK geriatric medicine is predominantly a hospital-based specialty is open to challenge. Nevertheless, several other professional perspectives were included and it is likely that this provided some balance. The break in the Delphi exercise, due to COVID-19, is unusual and could be seen as compromising the findings. However, we updated the underpinning literature review to ensure that the proposals put forward in the first Delphi round remained evidence-based, and we reintroduced all competencies agreed in wave 1 of the Delphi into wave 2 to compensate for this. The fact that the work was completed after the pandemic means that the competencies presented here have accommodated the substantial changes experienced in long-term care homes as a consequence of COVID-19.

In conclusion, we have derived a series of competencies which outline the expertise required to deliver good healthcare in care homes. These competencies underline how complex and technical medical care in this setting can be and could provide impetus to discussions about how training for doctors who provide such care is designed. The work will be of interest in those countries where care home medicine is not currently recognised as a specialty or where it is a contested responsibility between multiple specialties. This, in turn, could influence ongoing discussion about how healthcare delivery in care homes is structured.

## Supplementary Material

aa-23-1416-File002_afad237

## References

[1] Competition and Markets Authority . Care Home Market Study: Summary of the Final Report. 2017. Crown Office. London. Available online at: https://www.gov.uk/government/publications/care-homes-market-study-summary-of-final-report/care-homes-market-study-summary-of-final-report (30 July 2023, date last accessed)

[2] Gordon AL , Franklin M, Bradshaw L, Logan P, Elliott R, Gladman JRF. Health status of UK care home residents: a cohort study. Age Ageing 2014; 43: 97–103. 23864424 10.1093/ageing/aft077 PMC3861334

[3] NHS England . New care models: the framework for enhanced health in care homes, available online at https://www.england.nhs.uk/community-health-services/ehch/ (last accessed 30 July 2023).

[4] Chadborn NH , Goodman C, Zubair M et al. Role of comprehensive geriatric assessment in healthcare of older people in UK care homes: realist review. BMJ Open 2019; 9: e026921. 10.1136/bmjopen-2018-026921. PMC6500328 30962238

[5] O’Neill D , Briggs R, Holmerová I et al. COVID-19 highlights the need for universal adoption of standards of medical care for physicians in nursing homes in Europe. Eur Geriatr Med 2020; 11: 645–50. 32557250 10.1007/s41999-020-00347-6 PMC7298916

[6] Koopmans RTCM , Lavrijsen JCM, Frank HJ et al. Dutch elderly care physician: a new generation of nursing home physician specialists. J Am Geriatr Soc 2010; 58: 1807–9. 20863347 10.1111/j.1532-5415.2010.03043.x

[7] Royal College of General Practice . The GP Curriculum, available online at: https://www.rcgp.org.uk/mrcgp-exams/gp-curriculum (30 July 2023, date last accessed)

[8] Joint Royal Colleges of Physicians Training Board . The specialist curriculum for geriatric medicine, available online at: https://www.jrcptb.org.uk/specialties/geriatric-medicine (30 July 2023, date last accessed)

[9] Ruaux S , Chadborn N. A qualitative exploratory study of training requirements for general practitioners attending older people resident in care homes. J Integr Care 2023; 31: 64–74.

[10] McCarthy L , Gordon A, Carroll R, Blundell A, Chadborn N. Developing core competencies in care home medicine for deployment in the U.K. Open Science Framework. 2022. Available from: https://osf.io/kjtb9 (30 July 2023, date last accessed)

[11] Tricco AC , Lillie E, Zarin W et al. PRISMA Extension for Scoping Reviews (PRISMAScR): checklist and explanation. Ann Intern Med 2018; 169: 467–73. 30178033 10.7326/M18-0850

[12] General Medical Council . Generic Professional Capabilities Framework. 2017. Crown Office. London. Available online at: https://www.gmc-uk.org/education/standards-guidance-and-curricula/standards-and-outcomes/generic-professional-capabilities-framework (30 July 2023, date last accessed).

[13] Bloom BS . Taxonomy of Educational Objectives: Cognitive and Affective Domains. New York: David McKay, 1956.

[14] Pangaro L , ten Cate O. Frameworks for learner assessment in medicine: AMEE guide no. 78. Med Teach 2013; 35: e1197–210. 23676179 10.3109/0142159X.2013.788789

[15] Goldberg SE , Cooper J, Blundell A, Gordon AL, Masud T, Moorchilot R. Development of a curriculum for advanced nurse practitioners working with older people with frailty in the acute hospital through a modified Delphi process. Age Ageing 2016; 45: 48–53. 26764394 10.1093/ageing/afv178

[16] Roller-Wirnsberger R , Masud T, Vassallo M et al. European postgraduate curriculum in geriatric medicine developed using an international modified Delphi technique. Age Ageing 2019; 48: 291–9. 30423032 10.1093/ageing/afy173 PMC6424375

[17] American Medical Directors Association . Core curriculum on medical direction in post-acute and long-term care. Available online at: https://apex.paltc.org/page/core-curriculum-on-medical-direction (30 July 2023, date last accessed).

[18] Robbins I , Gordon A, Dyas J, Logan P, Gladman J. Explaining the barriers to and tensions in delivering effective healthcare in UK care homes: a qualitative study. BMJ Open 2013; 3: e003178. 10.1136/bmjopen-2013-003178. PMC3717448 23872297

[19] Achterberg WP , Everink IH, Van Der Steen JT et al. We’re all different and we’re the same: the story of the European nursing home resident. Age Ageing 2019; 49: 3–4. 31838507 10.1093/ageing/afz145

[20] Price DW , Wagner DP, Kevin Krane N et al. What are the implications of implementation science for medical education? Med Educ Online 2015; 20: 27003. 10.3402/meo.v20.27003. PMC4409632 25911282

[21] Grealish L , Todd JA, Krug M, Teodorczuk A. Education for delirium prevention: knowing, meaning and doing. Nurse Educ Pract 2019; 40: 102622. 10.1016/j.nepr.2019.102622. 31521042

[22] Fisher JM , Gordon AL, Maclullich AMJ et al. Towards an understanding of why undergraduate teaching about delirium does not guarantee gold-standard practice—results from a UK national survey. Age Ageing 2015; 44: 166–70. 25324329 10.1093/ageing/afu154 PMC4601531

[23] Forrester-Paton C , Forrester-Paton J, Gordon AL et al. Undergraduate teaching in geriatric medicine: mapping the British Geriatrics Society undergraduate curriculum to Tomorrow’s Doctors 2009. Age Ageing 2014; 43: 436–9. 24610864 10.1093/ageing/afu024

[24] Stanyon MR , Goldberg SE, Astle A, Griffiths A, Gordon AL. The competencies of registered nurses working in care homes: a modified Delphi study. Age Ageing 2017; 46: 582–8. 28064168 10.1093/ageing/afw244 PMC5859996

