# Supplementary material for: Developing a list of core competencies for medical aspects of healthcare delivery in care homes: scoping review and Delphi process
Source: Age Ageing. 2023 Dec 28;52(12):afad237. doi: 10.1093/ageing/afad237 (PMC10756078; doi:10.1093/ageing/afad237)
Supplement: aa-23-1416-File002_afad237 [file aa-23-1416-file002_afad237.docx]

Developing a list of core competencies for medical aspects of healthcare delivery in care homes: scoping review and Delphi process

## Appendix 1 – PRISMA-ScR checklist

| **SECTION** | **ITEM** | **PRISMA-ScR CHECKLIST ITEM** | **REPORTED**  **ON PAGE #** |
| --- | --- | --- | --- |
| **TITLE** | | | |
| Title | 1 | Identify the report as a scoping review. |  |
| **ABSTRACT** | | | |
| Structured summary | 2 | Provide a structured summary that includes (as applicable): background, objectives, eligibility criteria, sources of evidence, charting methods, results, and  conclusions that relate to the review questions and objectives. | Page 1 - abstract |
| **INTRODUCTION** | | | |
| Rationale | 3 | Describe the rationale for the review in the context of what is already known. Explain why the review  questions/objectives lend themselves to a scoping review approach. | Page 2 background |
| Objectives | 4 | Provide an explicit statement of the questions and objectives being addressed with reference to their key elements (e.g., population or participants, concepts, and context) or other relevant key elements used to  conceptualize the review questions and/or objectives. | Page 3 methods |
| **METHODS** | | | |
| Protocol and registration | 5 | Indicate whether a review protocol exists; state if and where it can be accessed (e.g., a Web address); and if available, provide registration information, including the registration number. | Page 2 methods |
| Eligibility criteria | 6 | Specify characteristics of the sources of evidence used as eligibility criteria (e.g., years considered, language,  and publication status), and provide a rationale. | Page 3 methods |
| Information sources* | 7 | Describe all information sources in the search (e.g., databases with dates of coverage and contact with authors to identify additional sources), as well as the date the most recent search was executed. | Page 3 methods |
| Search | 8 | Present the full electronic search strategy for at least 1  database, including any limits used, such that it could be repeated. | Appendix 2 |
| Selection of sources of evidence† | 9 | State the process for selecting sources of evidence (i.e., screening and eligibility) included in the scoping review. | Page 3 methods |
| Data charting process‡ | 10 | Describe the methods of charting data from the included sources of evidence (e.g., calibrated forms or forms that have been tested by the team before their use, and whether data charting was done independently or in duplicate) and any processes for obtaining and  confirming data from investigators. | Page 3 methods |
| Data items | 11 | List and define all variables for which data were sought and any assumptions and simplifications made. | Page 3 |
| Critical appraisal of individual sources of evidence§ | 12 | If done, provide a rationale for conducting a critical appraisal of included sources of evidence; describe the  methods used and how this information was used in any data synthesis (if appropriate). | Page 3 methods |
| Synthesis of results | 13 | Describe the methods of handling and summarizing the data that were charted. | Page 3 |

| **RESULTS** | | | |
| --- | --- | --- | --- |
| Selection of sources of evidence | 14 | Give numbers of sources of evidence screened, assessed for eligibility, and included in the review, with  reasons for exclusions at each stage, ideally using a flow diagram. | Fig 1 - PRISMA  diagram |
| Characteristics of sources of evidence | 15 | For each source of evidence, present characteristics for which data were charted and provide the citations. | Page 6 - results |
| Critical appraisal within sources of evidence | 16 | If done, present data on critical appraisal of included sources of evidence (see item 12). | Page 6 - results |
| Results of  individual sources of evidence | 17 | For each included source of evidence, present the  relevant data that were charted that relate to the review questions and objectives. | Page 6 results |
| Synthesis of results | 18 | Summarize and/or present the charting results as they relate to the review questions and objectives. | Appendix 3; Ap |
| **DISCUSSION** | | | |
| Summary of evidence | 19 | Summarize the main results (including an overview of concepts, themes, and types of evidence available), link to the review questions and objectives, and consider the relevance to key groups. | Page 13, discussion |
| Limitations | 20 | Discuss the limitations of the scoping review process. | Page 14, discus |
| Conclusions | 21 | Provide a general interpretation of the results with respect to the review questions and objectives, as well  as potential implications and/or next steps. | Page 14/15, discussion |
| **FUNDING** | | | |
| Funding | 22 | Describe sources of funding for the included sources of evidence, as well as sources of funding for the scoping  review. Describe the role of the funders of the scoping review. | Page 15 - funding statement |

## Appendix 2- Exemplar Search String from Medline

1 Competency-Based Education/

2 Clinical Competence/

3 "core competencies".mp.

4 Education, Medical, Graduate/ or Education/

5 Educational Measurement/

6 Curriculum/ or curricul*.mp.

7 Teaching/ 161428

8 1 or 2 or 3 or 4 or 5 or 6 or 7

9 "care home".mp.

10 Nursing Homes/

11 Homes for the Aged/

12 Long-Term Care/

13 Residential Facilities/

14 9 or 10 or 11 or 12 or 13

15 aged/ or "aged, 80, and over"/ or frail elderly/

16 14 and 15

17 postgraduate.mp.

18 Physicians/

19 Geriatrics/

20 Health Services for the Aged/ or gerontology.mp.

21 17 or 18 or 19 or 20

22 8 and 16 and 21

## Appendix 3 – Competencies extracted from literature review categorised against the General Professional Capabilities Framework Domains

| Adapted GPCF Domains | Competencies Required of Postgraduate Doctors in the Care Home Setting |
| --- | --- |
| **1. Professional Values and Behaviour -** *Demonstrate appropriate personal and professional values and behaviours. Acting with honesty and integrity, treating all with respect and acting in a professional manner in all circumstances, whilst being able to monitor own behaviour, knowledge and limitations by self - assessment.* | - Recognize that the role of the doctor within care homes is not limited to clinical management and includes providing advice to homes on medical-related aspects of leadership, administration, education, quality improvement and health policy. - Appraise knowledge and expertise continuously to maintain knowledge of the changing social, regulatory, political and economic factors that affect medical and health services of care home patients. - Describe how the experiences and views of individual doctors may influence their perspectives, presentation of issues, and care provision. - Demonstrate recognition and respect for the diversity of the older population, including physiological function, health status, belief systems, cultural and ethnic backgrounds, values and personal preferences, to therefore provide a holistic approach to care drawing on physical, psychosocial and spiritual factors. - Demonstrate a leadership role in developing the culture within the care home to support effective healthcare delivery to residents. - Recognize the importance of prioritising care home workload within competing clinical priorities. - To serve as an advocate for patients and their caregivers with regards to healthcare issues within the care home and to provide advice on medical and patient care issues at a facility level. |
| **2. Professional skills: incorporating**  2a Practical Skills - *Being able to accurately record and convey information, either verbal or written. Understanding medical forms or certifications and being able to effectively use IT.* | - Work with the staff in the care home to define how medical aspects of care will be documented and shared by doctors and care home staff. - Appreciate and demonstrate the use of technological advances utilised in the care of older adults in care homes including telephone advice, telemedicine and telecare. - Describe the interface between primary care and specialist geriatric care. - Assemble documentation to ensure a complete and accurate history is recorded and that relevant updates are added. - Describe how and where to find the evidence required to complete a comprehensive medical assessment on care home patients, recognising that their care may have been fragmented when delivered outside of the care home - Demonstrate comprehensive completion of documentation regarding transfer, discharge and death and that it is available in a timely manner to ensure continuity of care.   **Transfer of Information and Patients**   - Evaluate the transfer of patients to the hospital from the care home, ensuring the caretaking team has correct information on the acute events necessitating transfer, goals of transfer, medical history, medications, allergies, baseline cognitive and functional status, advance care plan and responsible primary care provider. - Identify ways to simplify and ensure the flow of information between hospital, care home, home health, hospice and the doctors’ office to assure continuity of patient care throughout the long term care continuum. - Appreciate the doctor’s role in transitions of care and knowledge of specific transfer documents. - Describe strategies to minimize inappropriate hospitalization and improve transition to and from the hospital. |
| 2b Communication and Interpersonal Skills - *High level communication with patients, relatives, carers, guardians and others by: establishing a respectful doctor-patient relationship and allowing for shared and supportive discussion and decision making with patient consent. Being aware of communication difficulties and cultural differences.* | - Demonstrate the ability to lead multidisciplinary team discussions at team and family meetings where it is appropriate for the doctor to do so. - Identify and assess barriers to communication such as hearing and/or sight impairments, speech difficulties, aphasia, limited health literacy and cognitive disorders. When present, demonstrate the ability to use adaptive equipment and alternative methods to communicate (e.g. with the aid of family/friend, caregiver.) - Analyse uncomfortable feelings when discussing medical decisions with families and patients and ensure that ethical and professional concerns are dealt with, prior to difficult conversations. - Execute effective communication with patients, the patients' family, care staff, and others, using interpersonal and communication skills, and assist the facility in developing communication systems, where relevant to the delivery of medical care. - Address older adults in the context of their unique situations (e.g. considering family, religion, culture, finances, values, cognitive status, sensory impairments and living arrangements). - Discuss and determine appropriate goals of care with patients and their families by communicating sensitively and ensuring shared decision making, respecting the patient’s autonomy at all times but especially when language barriers are present. |
| 2c. Dealing with Complexity and Uncertainty - *Show appropriate professional behaviour and judgement in a range of clinical and non-clinical circumstances. Adapting to responsiveness to treatment and ensure humane intervention where deemed necessary. Ethical issues must be identified and discussed.* | - Determine whether an older patient has sufficient mental capacity to give an accurate history, make decisions and participate in developing the medical plan of care. - In patients with life-limiting or severe chronic illness, assess pain and distressing non-pain symptoms (dyspnoea, nausea, vomiting, and fatigue) at regular intervals and provide appropriate treatment according to their goals of care. Identify with the patient, family and care team, when goals of care and management should transition from curative, to maintenance or palliative comfort care. - Describe and manage behaviours which challenge and psychological symptoms of dementia. Recognize that physical, chemical or informal restraint represent poor practice and so be able to describe alternative approaches to management. - Examine and establish planning for end of life issues including; advance care directives, do not resuscitate orders, hospice transfer, living wills, and lasting power of attorney for health care. This should be done in conjunction with the patient's and family’s preferences. - Demonstrate an approach to care that combines chronic and acute elements. In addition to attention to apparent and difficult to recognize acute or subacute problems (e.g. delirium), attention must also be directed to chronic problems. - Describe how multiple conditions and fluctuating clinical status are managed in parallel and how conditions requiring specific medical intervention can be identified. |
| 2d. Clinical Skills **-** *Demonstrate logical and comprehensive history taking, diagnosis, medical management, use of medical devices, health promotion, disease prevention and infection control.* | - Analyse and appreciate the differences in medical treatment for the older adults with frailty, especially those with chronic and incurable conditions compared to other patients in primary care practice.   **Assessment and Screening**   - Appropriately administer and interpret the results of standardized screening and assessment tools for functional assessment, specifically concerning; affect, alcohol abuse, anxiety and sleep, agitation and behaviour which challenges, cognition and mental status, constipation, delirium, dementia, depression, gait, mobility and risk of falls, hydration, language, nutrition, physical function, pain, pressure ulcers, speech and communication, urinary or faecal incontinence, visual and hearing impairment and wounds. - Conduct comprehensive geriatric assessment (CGA) for a care home patient.   **Geriatric syndromes**   - Define the diagnosis, pathophysiology, management and preventative strategies for specific disease processes and geriatric syndromes: anorexia, psychosis, depression, delirium, anxiety and apathy, sensorium, parkinsonism, injury, tremors, musculoskeletal problems, continence, pressure ulcers, iatrogenic illness, cerebrovascular disease and stroke, deep vein thrombosus, cardiac abnormalities and congestive heart failure, rheumatoid conditions, osteoporosis, diabetes mellitus in older people, chronic obstructive pulmonary disease, malnutrition, dehydration and infections. - Describe and evaluate a patient presenting with the following symptoms: breathlessness, weight loss, nausea, dizziness, voiding difficulties, tiredness, cardiac arrhythmia, oedema, behaviours which challenge including both verbal and physical agitation, weakness, gait abnormality and pain.   **Dementia**   - Define the diagnosis, pathophysiology, preventative strategies and management for dementia and behaviours which challenge.   **Frailty**   - Identify frailty using standard validated assessments and manage frailty syndromes and conditions that might exacerbate frailty e.g. anaemia, polypharmacy   **Falls**   - Evaluate mobility and identify strategies for the prevention of falls including physical therapy. Define predisposing factors such as; delirium, polypharmacy, orthostatic hypotension and food associated hypotension, altered leg strength and balance. Detect osteoporosis and identify it as a precursor to hip fractures, especially within the first 3 months of care home admission.   **Nutrition**   - Evaluate nutritional status and conduct assessments of nutritional needs and treatment of malnutrition and dehydration. Appropriately use oral supplements and parenteral feeding tubes and understand weight loss causes including: medications, emotional, alcoholism, late life paranoia, swallowing difficulties, oral factors, nosocomial infections, dementia, hyperthyroidism, enteral problems, diet and stones, cancer.   **Medical Devices**   - Be able to provide advice and support decision-making in managing catheters and feeding tubes in the care home setting.   **Infection control**   - Advise on care-home specific infection control issues and approve specific infection control policies to be incorporated into facility policies and procedures, such as immunization.   **Rehabilitation**   - Assess and identify conditions, impairments, disabilities and excess disabilities that may be amenable to rehabilitation alongside therapy professionals weighing up risks and benefits. Then design and implement effective rehabilitation plans, striving for functional independence, based on the patient’s health status, wishes and tolerability.   **Prescribing Medicines Safely**   - Describe the specific challenges and opportunities of using therapeutic agents in the care home setting including route of administration, supervision of administration, covert administration and monitoring of effects of therapeutic intervention. - Recognize common side effects in light of age-related changes in renal and hepatic function, body composition, central nervous system sensitivity and other comorbidities. Also recognize the effect of functional status and that other medications (drug- drug interactions) can cause adverse drug reactions. - Review medication for continued efficacy and presence of side effects and to actively reduce polypharmacy. This will involve gathering information prior to drug administration for baseline values and then monitoring for current drug responses, patient allergy review, clinical and laboratory monitoring, achievement of therapeutic goals and predicting adverse effects and unpredictable effects. |
| **3. Professional Knowledge, incorporating:**  *Describe legislative requirements and health services under the UK healthcare system.* | - Describe the regulatory and governance frameworks for care homes where they relate to delivery of medical care. - Describe the services provided within the care home and institutional care settings, give examples of the different practice models and define long term care. - Describe how the administrative structure of care homes, including the respective roles of the registered owner and manager, can influence delivery of medical care. - List basic aspects of long-term care and care home funding. |
| **5. Capabilities in Leadership and Team Working -** *Awareness of leadership responsibilities as a clinician and appreciation of working as a team and the roles within the team.* | - Coordinate and effectively participate in inter-professional, team-based approach to providing care. - Describe the roles and involvement of care home staff (attending doctors, physician associates, consultants, care assistants, practice nurses, nurse practitioners, dieticians, and therapists. - Establish the team’s mission, values and goals, understand group dynamics, and develop key interpersonal and communication skills that facilitate working effectively as member of an inter-professional health care team (e.g. role negotiation, conflict management, problem solving, listening to perspectives of stakeholders and decision making). |
| **6. Capabilities in Patient Safety and Quality Improvement -** *Acknowledge risks to patients and strategies to keep patients safe.* | - Review, respond to, and participate in required external surveys and inspections of the care home facility. - Recognize health care system issues that negatively affect care and failure to thrive, including maintenance of function and health, environment and maintenance of resident dignity. - Reduce iatrogenic events among residents in all settings through implementation of patient specific and system wide strategies to prevent; falls, immobility, injuries, delirium, pressure ulcers, incontinence, malnutrition, dehydration, indwelling catheter use, nosocomial infections, deep vein thrombi, restraints, depression, and functional decline, all achieved through interdisciplinary involvement and doctor knowledge. - Demonstrate knowledge of and evaluate commonly accepted quality indicators and participate in continuous quality improvement and culture change for older adults. - Describe how a home-like environment can be maintained in the midst of complex medical care in a care home setting. |
| **7. Capabilities in Safeguarding Vulnerable Groups -** *Recognize and accept responsibility of safeguarding.* | - Describe how a doctor can work with care home staff to prevent, recognise, respond to and report elder abuse in all its forms. - Identify older persons at high safety risk, in particular unsafe drivers or those experiencing elder abuse/neglect/social deprivation and develop a plan that allows maximum control of the patient. |
| **8. Capabilities in Education and Training -** *Safely supervise and develop learner’s knowledge, skills and attitudes. Promote inter-professional learning and reflect on effectiveness of educational activities.* | - Demonstrate the ability to teach patients, caregivers and others and promote a practise-based learning culture within the care home with effective supervision and periodic evaluation of care home specific administrative and clinical performance. - Describe and teach current theories of ageing, including social, psychological and biological. |
| **9. Capabilities in Research and Scholarship -** *Keep up to date with current literature, guidelines and trials.* | - Describe where to find and access Continuing Professional Development CPD, related to care homes. |

## Appendix 4 – Competencies and Domains Carried into first round of Delphi exercise

| **Domain** | Competencies |
| --- | --- |
| **1. History taking and communication with residents** | Can make use of recognised consultation techniques such as The Pendleton Model to take an appropriately focused history to include residents with complex multiple comorbidities as well as using relatives and care staff as informants if needed. |
|  | Can communicate complex problem lists and management plans without using medical jargon to ensure residents, family and carers understand. |
|  | Can communicate effectively with people with cognitive impairment and conduct triadic consultations ie doctor – patient – carer |
|  | Can communicate effectively around sensitive topics such as advance care planning |
|  | Can communicate effectively with residents with cognitive impairment/dementia / families |
|  | To be able to effectively incorporate handovers from care home staff to develop a working patient history |
| **2. Clinical Skills** Assessment and planning | Can target history-taking to discriminate between likely diagnoses as part of a comprehensive approach to medical assessment. |
|  | Can appropriately prioritise acute conditions over less urgent clinical problems. |
| Comprehensive assessment- | Can appropriately administer and interpret the results of standardized screening and/or comprehensive assessment for:   - affect - anxiety - behaviours which challenge - bowel and bladder function - cognition - delirium - depression - gait and balance - hearing - nutrition - pressure ulcers - sleep - vision - breathlessness - loneliness |
| Medical devices | Can provide advice and support decision-making around artificial nutrition and syringe drivers in the care home setting. |
| **3. Knowledge of ageing** | can demonstrate knowledge of:   - theories of ageing - the functional, physical, cognitive, psychological social and spiritual changes common in older age - frailty - the ageing population and demographics - comprehensive geriatric assessment - non-specific presentations of illness and infection |
|  |  |
|  |  |
|  |  |
|  |  |
|  |  |
| **4. Management of long-term conditions and comorbidities in the care home setting** | Can manage the following chronic conditions effectively:   - diabetes - dementia - depression - anxiety - psychiatric illnesses - Parkinsonism - Motor Neurone Disease - Multiple Sclerosis - Stroke - COPD - Heart Failure - Osteoarthritis - Osteoporosis - Ischaemic Heart Disease - Frailty - Musculoskeletal problems - Multi-morbidity - Pain - Malignant disease - Renal Disease |
| **5. Dementia and behaviour management** | Can assess and manage residents with dementia including:   - Cognitive Status Assessment - Behavioural and Psychological Symptoms associated with Dementia - Effect of dementia on management of comorbidities - Knowledge of the commonly used drug treatments for dementia - Application of the Mental Capacity Act in clinical practice |
|  | Ability to communicate appropriately with family |
|  | Ability to assess capacity to give informed consent |
|  | Ability to understand best interests and make appropriate decision making on behalf of families and carers |
|  | Knowledge and clinical expertise in palliative and end of life care in dementia |
| **6. Falls** | Can assess and manage older patients presenting with falls. Including:   - Knowledge of interventions for fall prevention - How to take a history for falls and examine for common causes of falls - The ability to perform clinical assessment of injuries to identify residents in need for further investigation/imaging. - Diagnosis and management of osteoporosis |
| **7. Continence** | Can assess and manage urinary and faecal incontinence. |
|  | Knows how and when to refer for further specialist advice (E.g. continence nurse specialist, OT), appreciating that referral pathways will differ locally. |
|  | Can provide advice and support decision making in the management of urethral, suprapubic catheters, and urethral sheaths, as well as knowing when to insert and when to initiate a trial without catheter. |
|  | To provide advice on best methods for continence optimization and preservation |
| **8. Nutrition** | Can assess the nutritional status of older people |
|  | Can work with other health professionals to devise appropriate nutritional support strategies for residents. |
|  | Can modify nutritional approaches to take account of disease processes, tissue viability, recovery from illness and surgery. |
|  | Can describe and use common nutritional assessment tools |
| **9. Rehabilitation** | Knows about the international classification of functioning, disability and health and can classify care problems according this to help shape rehabilitation plans. |
|  | Can establish which disabilities may be amenable to rehabilitation alongside therapy professionals. |
|  | Can participate in the design and implementation of effective rehabilitation plans, based on patient’s state, wishes and tolerability. |
| **10. Management of acute conditions** | Can identify an acutely unwell patient and make decisions about escalation of treatment, including hospital transfer |
|  | Can recognise, diagnose and manage delirium in combination with non-specific presentations of illness presenting both acutely or sub-acutely. |
|  | Knowledge of monitoring acute conditions by making effective use of the National Early Warning Score |
| **11. End of life care** | Can provide care for the dying patient and his/her family, including:   - recognising when to discontinue investigations and treatment on an individualised, holistic and situationally appropriate basis - recognising when to prescribe anticipatory medications - being able to manage common ethical dilemmas in older patients with frailty, including resuscitation and escalation of treatment in people who lack mental capacity to make these decisions. - being able to describe and use care pathways to support care of the dying, such as the Gold Standards Framework - recognising when to seek specialist palliative care input |
| **12. Pain Management** | Knows how to assess pain in adults with frailty and cognitive impairment |
|  | Ability to modify pain management strategies in residents with communication difficulties and cognitive impairment to include:   - non-pharmacological strategies - modifications to common analgesic regimens |
|  | To be aware of appropriate delivery mechanisms for analgesia |
|  | Understand and know how to assess distress in people with dementia or communication difficulties e.g. stroke |
|  | To be able to distinguish between malignant and non-malignant pain, prescribing use of regular (rather than PRN) analgesia for patients who will not prompt for pain relief |
| **13. Therapeutics and safe prescribing** Pharmacology | Can prescribe and deprescribe competently in older adults including:   - describing changes in the pharmacodynamics and pharmacokinetics of commonly used drugs within the older population - Recognising the importance of drug-patient and drug-drug interactions in this cohort. - Make appropriate prescribing and de-prescribing decisions, including the use of structured tools (e.g. STOPP/START) when appropriate. - Knowledge of clinical consequences of polypharmacy in the care home setting - Describing ways to support concordance in the older population - Knowledge of covert administration of medication in patients without mental capacity - Able to advise on the appropriate use of crushed, dispersible and liquid medications in residents with swallowing problems |
|  | To be able to communicate key information on risks/adverse outcomes from prescribing to staff without nursing or medical qualifications |
|  | To understand that a range of prescribing and dispensing processes in care homes may be useful such as centralised pharmacy facilities, and understand the influences on prescribing choices and speed of access to medications as a result of this |
| **14. Communication and cooperation with colleagues** | Communicate around complex transfers of care with professionals of multiple disciples with varying levels of experience to include therapists, doctors, nurses, other allied health professionals and professional caregivers based both in the community and in hospital with pertinent and timely clinical information to enable appropriate investigation and management. |
|  | Can communicate with staff without nursing qualifications, maintaining confidentiality |
| **15. Working Collaboratively with Care Homes** | Able to describe the staffing structures within modern care homes and the competencies held by different care homes staff groups |
|  | Able to recognise the complementary roles of health and social care staff in providing healthcare and care homes |
|  | Can describe the ways in which doctors can support care home teams to deliver routine aspects of healthcare |
| **16. Teaching and training** | Able to modify teaching to meet the needs of staff with diverse educational and professional backgrounds to include care staff without nursing and medical qualifications |
| **17. Quality improvement and evidence based practice** | Able to find and interpret current best-evidence in long-term care |
|  | Able to lead and support quality improvement in the care home sector, working with staff from multiple backgrounds. |
|  | Able to recognise the barriers and challenges to quality improvement in the care home context |
| **18. Capabilities in Safeguarding Vulnerable Groups** | Able to recognize and respond to elder abuse including physical, psychological, emotional, financial, sexual and institutional abuse, whether by omission or commission. |
|  | Able to describe safeguarding procedures and adhere to these in patient care |
| **19. Leadership team working** | Able to listen, support and advise a team of professionals and care staff with diverse clinical and educational backgrounds. |
| **20. Infection control** | Can describe the specific challenges of infection control in the care home sector. |
|  | Can describe how to manage communicable diseases to include diarrhoea/vomiting, scabies and influenza in the care home sector. |
|  | Can describe prevention vaccination strategies required to support health and wellbeing in the care home sector. |
| **21. Legal Framework** | Can describe the care home specific considerations with regard to:   - death certification and the role of the Coroner/Procurator Fiscal - the mental health act - The mental capacity act - The Continuing Care Assessment process - Deprivation of Liberty Safeguards - Confidentiality - advanced directives and living wills - Lasting power of attorney - decisions regarding resuscitation - communicable disease notification - medical risk and driving - the funding of long-term care and the legal obligations of NHS providers to care home residents |
